# Dependence of model-based RSA accuracy on higher and lower implant surface model quality

**DOI:** 10.1186/1475-925X-12-32

**Published:** 2013-04-16

**Authors:** Frank Seehaus, Judith Emmerich, Bart L Kaptein, Henning Windhagen, Christof Hurschler

**Affiliations:** 1 Laboratory for Biomechanics and Biomaterials, Department of Orthopaedics, Hannover Medical School, Anna-von-Borries-Str. 1-7, Hannover, 30625, Germany; 2 BG Unfallklinik Duisburg, Department of Trauma Surgery, Großenbaumer Allee 250, Duisburg, D-47249, Germany; 3 Department of Orthopaedics and Division of Image Processing, Department of Radiology, Leiden University Medical Center, PO Box 9600, Leiden, 2300 RC, The Netherlands

**Keywords:** Roentgen stereophotogrammetric analysis, Migration, Reversed engineering models, Accuracy, Precision

## Abstract

**Background:**

Model-based Roentgen Stereophotogrammetric Analysis (MBRSA) allows the accurate *in vivo* measurement of the relative motion between an implant and the surrounding bone (migration), using pose-estimation algorithms and three dimensional geometric surface models of the implant. The goal of this study was thus to investigate the effect of surface model resolution on the accuracy of the MBRSA method.

**Methods:**

Four different implant geometries (knee femoral and tibial components, and two different hip stems) were investigated, for each of which two reversed engineering (RE) models of differing spatial digitizing resolution were generated. Accuracy of implant migration measurement using MBRSA was assessed in dependence on surface model resolution using an experimental phantom-model set up.

**Results:**

When using the lower quality RE models, the worst bias observed ranged from -0.048 to 0.037 mm, and -0.057 to 0.078 deg for translation and rotation respectively. For higher quality reverse engineering models, bias ranged from -0.042 to 0.048 mm, and -0.449 to 0.029 deg. The pair-wise comparisons of digitizing resolution (higher vs. lower quality) within the different implant type revealed significant differences only for the hip stems (p < 0.001).

**Conclusion:**

The data suggest that the application of lower resolution RE models for MBRSA is a viable alternative method for the *in vivo* measurement of implant migration, in particular for implants with non symmetrical geometries (total knee arthroplasty). Implants with larger length to width aspect ratio (total hip arthroplasty) may require high resolution RE models in order to achieve acceptable accuracy. Conversely, for some axis the bias for translation are clearly worse for translation, and are marginally better for rotations using the lower resolution RE models instead of the higher ones. However, performed box plots ranges were well within what has been reported in the literature. The observed lower accuracy and precision of the measurements for hip stem components for rotations about the superior-inferior direction is presumably the result of the nature of the MBRSA method. This well known effect within MBRSA for rotations about the axis of symmetry of axially-symmetric objects do not change the contour of the projected image to as large a degree as motion about a non-symmetric axes. It is not possible to detected this small motion as accurately using pose-estimation methods. This may affect the “higher” accuracy for the applied lower resolution RE models.

## Background

Roentgen Stereophotogrammetric Analysis (RSA) is a highly accurate method for the *in vivo* detection of musculoskeletal kinematics [1-4]. Continuous improvements in object recognition, mathematical as well as computer-graphics algorithms, have allowed the RSA method to find a wide range of applications within the field of orthopaedic research [5-13]. The RSA method remains of particular clinical importance, because it allows the measurement of implant migration in the first two postoperative years, which has been shown in long term clinical studies to correlate well with a later aseptic implant loosening [14,15]. Implant migration presents the three dimensional motion between an implant and its surrounding bone over a follow up period of two years in relation to the direct post-operative situation [16]. Furthermore, aseptic loosening remains a major problem associated with total joint arthroplasty [17-19] and RSA present the gold standard to quantify the implant fixation [17,20]. The power and clinical relevance of RSA is to investigate implant fixation within a relative short observation interval has been documented based on long-term studies [14,15].

Model-based RSA (MBRSA) is a method, utilizes bone markers as well as pose-estimation algorithms and three dimensional surface models of the implant to compute the *in vivo* migration of the implant [2,5,21]. To date, computer aided design (CAD) drawings or reverse engineering (RE) technologies have been used to obtain the necessary three dimensional surface models of the implants. To determine implant motion, a virtual contour of the three dimensional surface model of the implant is projected into the RSA-image pairs, and matched (fitted) against the actual contour of the implant, which is detected by means of the canny-operator edge detection algorithm [22]. The three dimensional surface model is thereby translated and rotated by the pose estimation algorithm until the best match (fit) between the actual and virtual contour is found [5,6,21]. Similar geometry-based methods have been previously developed for measurements of implant migration [8,23], as well as to investigate joint-kinematics by means of fluoroscopic image sequences [24-31].

The accuracy of RSA in general has been investigated in several experimental phantom-model studies, or by means of double (repeated) patient examinations during clinical application [5,6,8,9,11,12,21,32-36]. As has been previously stated by Ryd *et al.* (2000), “… the accuracy of RSA depends on a large number of factors including the radiographic equipment, the RSA set-up, the number of markers, size of and distance between marker configurations” [37]. This principal can be extended to MBRSA, in stating that accuracy in this case is also dependent on the quality of the geometric surface models used. A characterization of this effect is of interest because one application scenario which has been proposed is the integration of a RE scanning step into the implant manufacturing process for quality assurance purposes. Current RE technology has advanced to such a degree that this scenario could become reality. The cost of RE devices has dropped whilst the quality of the digitized surface models is improving and scanning time has been reduced. An additional digitizing step within the manufacturing process would, as a side-effect, provide accurate surface models which could further be used for MBRSA and may facilitate wider application of the method for standardized early preclinical studies as suggested by Valstar *et al.* (2005).

The influence of the source and mesh density of three dimensional geometric models (CAD, RE, number of triangles) on MBRSA accuracy has been previously investigated [5]. As a result, RE models take account of the highest degrees of manufacturing tolerances. Interchangeable applicability of MBRSA using RE models with the classical marker-based RSA method has been shown in previous studies [16,38,39] However, the effect of the digitization quality of the RE models for differing implant geometries has not. A characterization of this effect is of interest because one application scenario which has been proposed is the integration of a reverse engineering scanning step into the implant manufacturing process for quality assurance purposes. This is of interest, because the time required for lower resolution scanning is significantly less than for high resolution scanning. With the digitizing equipment available to us, the scanning time for one implant, which includes both the time for digitization and mesh generation, is approximately 120 minutes for the higher resolution device (i.e. ATOS II, GOM mbH, Braunschweig, Germany), and about 90 minutes for the lower resolution device (i.e. ATOS I, GOM mbH, Braunschweig, Germany). Acquisition time is thus an important parameter, especially when considering that some manufacturers envision scanning every manufactured prosthesis for MBRSA as well as quality assurance purposes. Current reverse engineering technology has advanced to such a degree that this scenario could become reality. The cost of reverse engineering devices has dropped whilst the quality of the digitized surface models is improving and scanning time has been reduced.

The goal of this study was thus to evaluate the effect of RE spatial digitizing resolution on the accuracy of MBRSA migration measurement in an experimental phantom-model set-up. We compared the two commercially available devices at our disposal, with slightly different volumetric point spacing, spatial resolution, and total acquisition times. We hypothesized that different spatial digitization resolutions do not affect the accuracy and precision of migration measurement using MBRSA, and that accuracy of the method using RE models attained using the two commercially available digitizing systems falls within the range of accuracy reported for marker-based RSA in the literature.

## Methods

**Figure 1:**
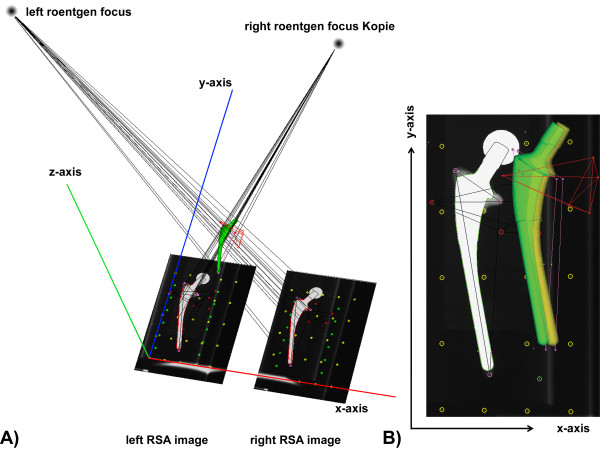
**Reference RSA radiograph. A)** Migration is represented with respect to a global fiducial coordinate system defined relative to the calibration box translation along the medio-lateral (x-axis, red) and superior-inferior (y-axis, blue) axes constitute in-plane motion, and translation about the posterior-anterior-axis (z-axis, green) out-of-plane motion; rotation about the posterior-anterior-axis (Rz-axis, green) further described in-plane motion, and about the medio-lateral (Rx-axis, red) and superior-inferior axes (Ry-axis, blue), out-of-plane motion respectively. **B)** The applied implant phantom-model enables the simulation of implant migration. This migration simulation was enabled by the micromanipulators integrated within the phantom model. The imposed set-point motion within the measurement protocol was 1.0 mm for translation about each axis, as well as 1.31 deg for out-of-plane (x-, y-axis) and 1.19 deg for in-plane (z-axis) rotational motion.

**Figure 2:**
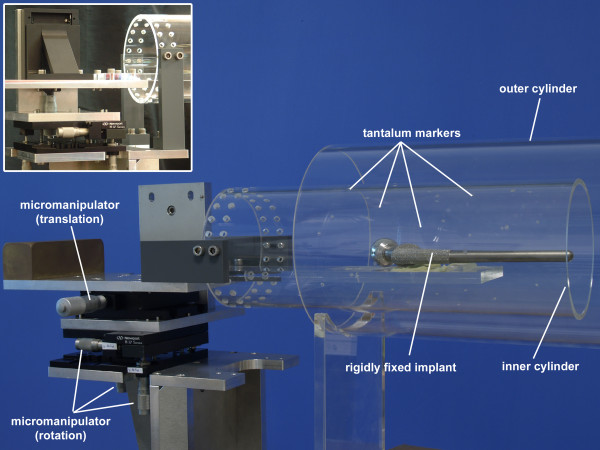
**Phantom Model.** The phantom model enables the migration simulation of the implant according to a zero relative prosthesis-bone motion and a relative prosthesis-bone motion protocol. For the relative prosthesis-bone motion protocol the inner cylinder must be rigidly fixed to the phantom model basic (image in upper left corner). The prosthesis components investigated were rigidly fixed onto the Plexiglas beam. This beam can be positioned within the Plexiglas tube using micromanipulators for translation and rotations. Three rotational and one translational manipulator were used, whereby the single translational manipulator was repositioned to allow translational motion about each of the three axes of motion investigated.

Lower resolution RE models (ATOS I) were compared with earlier obtained high resolution RE models (ATOS II) [38] using the same migration measurement protocol previously used (measurement set-up, phantom model). These RSA radiographs were analyzed a second time using lower resolution models. The images were generated within a uni-planar RSA measurement set-up, consisting of two synchronized analog roentgen tubes (Philips MCD 105 and Philips Medio 50 CP-H, Philips, Medical Systems GmbH, Hamburg, Germany) and a carbon-fiber calibration box (Medis Medical Imaging Systems bv, Leiden, Netherlands). A bone and implant phantom-model was rigidly attached to the calibration box. The bone and implant phantom-model enables the simulation of implant migration which was represented with respect to a global fiducial coordinate system defined relative to the calibration box (Figure 1). This phantom-model was constructed to enable the migration simulation of the implant according to two different protocols: zero relative prosthesis-bone motion, in which the prosthesis and bone are moved as one rigid body together, and relative prosthesis-bone motion, whereby the prosthesis is moved relative to the bone [38]. A Plexiglas tube was used to simulate the bone about the implant (Figure 2; inner cylinder). In order to simulate bone makers adjacent to the various implant geometries tested, 36 spherical tantalum markers of 1.0 mm diameter (Tilly Medical Products AB, Lund, Sweden) were inserted into the Plexiglas tube. A further Plexiglas tube was used to represent the soft tissue surrounding the bone (Figure 2, outer cylinder). The prosthesis components investigated were rigidly fixed onto the Plexiglas beam, which can be positioned within the Plexiglas tube using micromanipulators. Three rotational and one translational manipulator were used, whereby the single translational manipulator was repositioned to allow translational motion about each of the three axes of motion investigated. Accuracy of the translational (ThorLabs Inc. Europe, Karlsfeld, Germany) as well as the rotational manipulator (Newport GmbH, Darmstadt, Germany) was characterized using laser-interferometry. Average mean (bias) and standard deviation relative to set points were less than 0.005 ± 0.002 mm in translation, and 0.000 ± 0.007 deg in rotation (n = 10 repetitions). The Plexiglas tube representing bone, as well as the plate to which the prosthesis components were attached, were both rigidly fixed to the precision manipulators in the zero relative prosthesis-bone motion protocol (Figure 2). Motions about six degrees of freedom were thus imposed on the implant and bone attached rigidly to one another (hence the term “zero relative motion”). Since no true motion between both rigid bodies takes place, the set-point of measured migration should thus be exactly zero. In the relative prosthesis-bone motion protocol, the Plexiglas bone tube was rigidly fixed, whereby the implant was moved relative to the tube (hence the term “relative motion”).

### Investigated prosthesis designs and RE models

**Figure 3:**
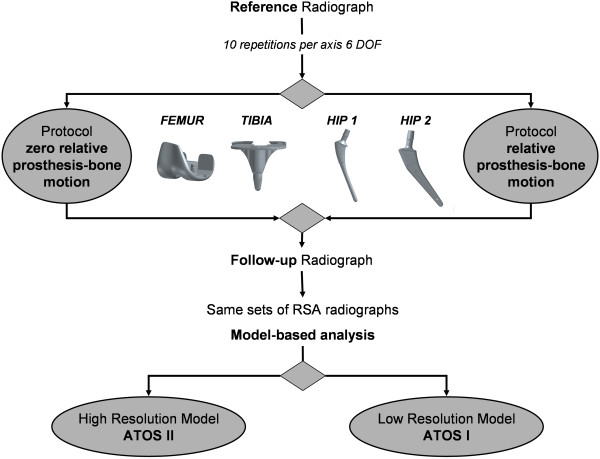
**Measurement protocol.** Schematic diagram showing the investigated implants, differing RE model quality, as well as the applied measurement protocols and analysis steps for each pair of RSA radiographs. Same sets of RSA radiographs were analyzed a second time, using lower resolution RE model. Only the high resolution RE model (ATOS II) of each investigated implant was replaced by lower resolution model (ATOS I) within analysis. The calibration settings bone-marker and actual contour detections within the pairs of RSA image remained unchanged from the first analysis using the high resolution models (ATOS II).

Four typical prosthetic components that varied in geometric design (Argomedical GmbH, Gifthorn, Germany) were investigated (Figure 3): one femoral (FEMUR) and one tibial (TIBIA) total knee arthroplasty paired-component, and two femoral total hip arthroplasty components, the Argo-TEP (HIP 1) and Antea (HIP 2). The geometric designs of the investigated components represent typical geometries for knee and hip total joint arthroplasty. Its basic geometric arrangement is similar to other prosthesis, especially in a typical a-p radiographic view. The both hip stem components of hip arthroplasty represents two typical design variations: one with a roundish long design in superior-inferior direction in an a-p radiographic view, the other with a flat design (expanded stem in medial-lateral direction) in the metaphyseal region. Individual RE models of the four implants were generated using two different optical non-contact fringe-projection digitizing systems (ATOS I and ATOS II, GOM, mbH, Braunschweig, Germany) [40], in order to generate RE models of differing quality.

**Figure 4:**
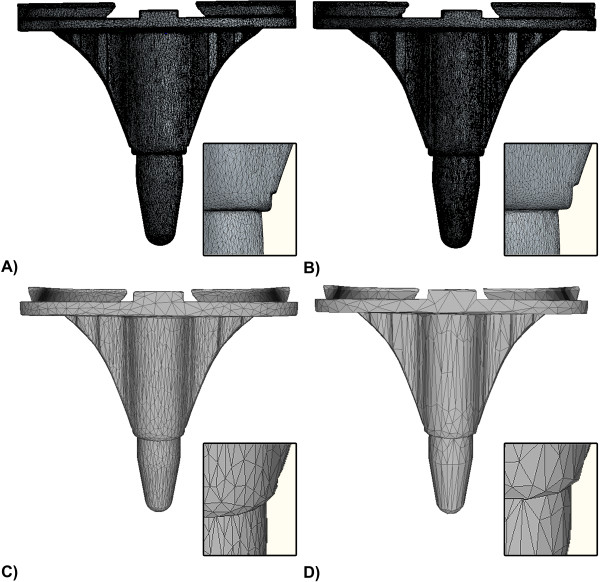
**RE surface model.** Surface model of a tibial total knee arthroplasty component generated with RE technology. Also visible is a close up of the tibial stem, in order to illustrate the quality of the mesh. **(A)** Surface model digitized using optical non contact digitizing system ATOS II, consisting of 113,934 points and 227,693 polygons. **(B)** Surface model digitized using optical non contact digitizing system ATOS I, consisting out of 103,188 points and 206,366 polygons. **(C)** Reduced ATOS II surface model (2,572 points and 4,999 polygons), and **(D)** reduced ATOS I model (2,502 points and 5,000 polygons).

The first set of models was generated with a volumetric point spacing of 0.08 mm and spatial resolution of 0.01 mm using the ATOS II system [38]. Additionally, the same prosthetic components were digitized twice with a volumetric point spacing of 0.125 mm and a spatial resolution of 0.02 mm using the ATOS I system. The number of digitized points of a scanned implant determines the number of polygons of the RE model representing the three dimensional surface in the raw scanned state (not optimized or reduced). RE models were generated which consisted of between 227,693 and 722,808 polygons (113,934 and 361,406 points) for the ATOS II, as well as between 142,874 and 356,225 polygons (71,439 and 178,136 points) for the ATOS I digitizing system. The raw meshes generated with the higher resolution ATOS II system were thus about twice as large in terms of the number of polygons as the lower resolution meshes. According to manufacturer suggestions at the time the investigations were performed, each RE model was subsequently reduced to 5,000 polygons for use in the pose-estimation algorithm within the MBRSA software (MBRSA 2.0 beta, MEDIS specials, Leiden, Netherlands). A quadric-based polygon surface simplification algorithm [41] (Figure 4) was used for polygon reduction, and the quality of the resulting surface models verified using mesh registration methods; a nominal-actual value comparison was performed between reduced and raw scanned state RE models using the ATOS Software v5.4 (ATOS Software v5.4, GOM mbH, Braunschweig, Germany). The difference between the reduced and the raw scanned meshes was ≤ ± 0.05 mm in all cases for the convex surface regions of the implant which contribute to the projected contours of the implant and are thus relevant for pose-estimation.

### Analysis

The same version of the MBRSA software package (MBRSA 2.0 beta, MEDIS specials, Leiden, Netherlands) and analysis protocol were used for the repeated analysis of the RSA radiographs. Only the RE model was replaced within each analysis: calibration settings including thresholds, bone-marker and actual contour detections within the pairs of RSA image remained unchanged from the first analysis [42,43], and thus do not contribute to the relative error between the analyses. Pose estimation of the replaced RE models were performed by allowing the same iterative inverse perspective matching (IIPM) algorithm [5] to run until the best fit was found, which was defined by the convergence of the difference parameter below a set value. The difference parameter was defined as the average of all deviations between both contours. A further stopping criterion intended to prevent run-out, was set to 50 iterations but never reached in any of the analyses performed.

### Statistical methods

Accuracy was expressed as the bias and precision of measured values of motion (x, y, z, Rx, Ry, Rz), whereby precision was expressed as the standard deviation (SD) of the repeated measures (n = 10 per axis). Bias was defined as the average difference between the measured and set-point values of motion. Set-point values were physically imposed using the micromanipulators as previously described [38]. The definitions of bias, precision, and “accepted reference value” reported herein are derived from and conform to the guidelines set forth in ASTM E-177-08. All computations were performed using SPSS (Version 13.0, SPSS Inc., Chicago, Illinois, USA). Box-plots were used to illustrate the variability of the data, and to facilitate the identification of possible measurement outliers and extreme values.

To compare migration measurement with the two digitizing resolutions and the four prostheses components investigated, a two-factor ANOVA (p < 0.05) was performed with two levels for the factor *digitizing resolution* (ATOS I, ATOS II), and four levels for the factor *implant type* (FEMUR, TIBIA, HIP 1, HIP 2). The dependent variables tested were the three components of implant translation and rotation respectively, which were measured (x, y, z, Rx, Ry, Rz). Where significant interactions between digitizing resolution and implant type were found, a simple main effects follow-up analysis was performed to compare the factor *implant resolution* within each level of the factor *implant type*.

## Results

**Figure 5:**
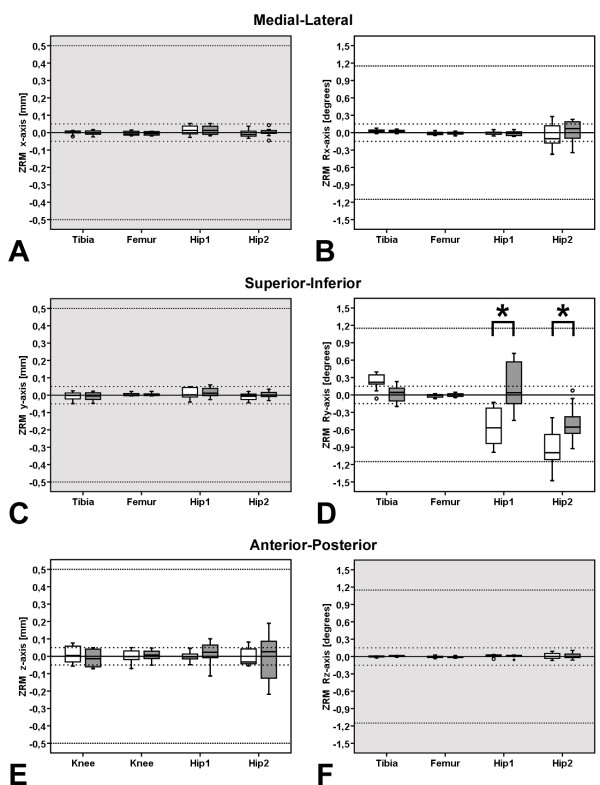
**Migration within zero relative prosthesis-bone motion protocol.** Box-Plots of migration measured using MBRSA and the zero relative prosthesis-bone motion protocol, the higher (ATOS II, white box) [38] and lower digitizing resolution RE models (ATOS I, gray box) are both shown. Gray background colored plots represents in-plane, white plots out-of-plane motion. The boxes bound the 25^th^ to 75^th^ percentile, the whisker bars show smallest and largest observe values. The horizontal line of the boxes represented median value, the dashed lines indicate bounding range of RSA accuracy reported in literature (*i.e.* between 0.05 to 0.5 mm, and between 0.15 to 1.15 deg) (Valstar et al. 2002, Kaptein et al. 2003). Migration is shown in (**A**) medio-lateral, (**C**) superior-inferior, and (**E**) anterior-posterior translation as well as for rotation about the (**B**) medio-lateral, (**D**) superior-inferior, and (**F**) anterior-posterior axes. Significant differences were calculated by two-factor ANOVA (p < 0.05), as well as the effects within a pairwise comparisons of the digitizing resolution in context to the dependent variables (x, y, z, Rx, Ry, Rz) were presented by a bold star on top of a bracket.

Using the zero relative motion protocol, significant effects for the factors digitizing resolution (p = 0.011) and the implant type (p < 0.001) were observed, as well as an interaction effect between these factors (p < 0.029). The follow-up pair-wise comparisons of digitizing resolution within the factor implant type revealed significant differences for HIP 1 (p < 0.001) and HIP 2 (p < 0.001, Figure 5).

**Figure 6:**
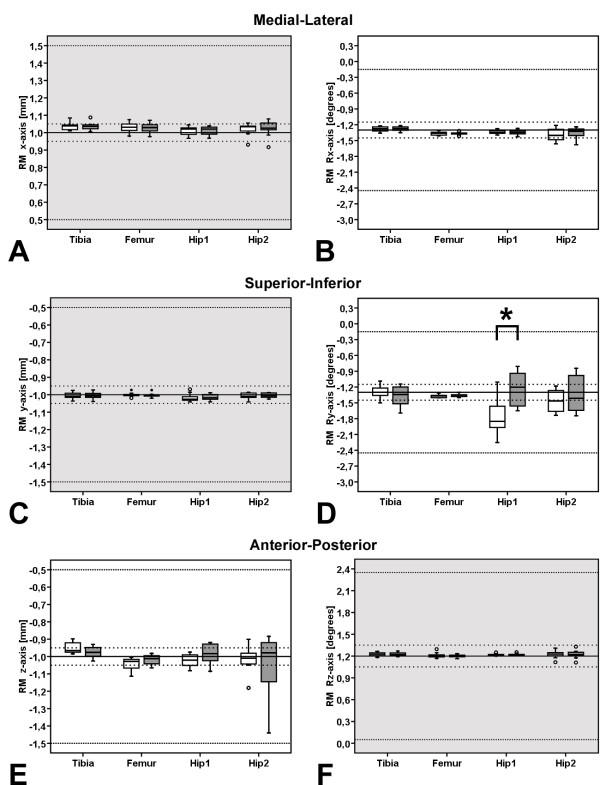
**Migration within relative prosthesis-bone motion protocol.** Box-Plots of migration measured using MBRSA and the relative prosthesis-bone motion protocol, the higher (ATOS II, white box) [38] and lower digitizing resolution RE models (ATOS I, gray box) are both shown. Gray background colored plots presenting in-plane, white colored out-of-plane motions. Migration is shown in (**A**) medio-lateral, (**C**)superior-inferior, and (**E**) anterior-posterior translation as well as for rotation about the (**B**) medio-lateral, (**D**) superior-inferior, and (**F**) anterior-posterior axes (see Figure 5 for a description of the box-plots). Significant differences were calculated by two-factor ANOVA (p < 0.05), as well as the effects within a pairwise comparisons of the digitizing resolution in context to the dependent variables (x, y, z, Rx, Ry, Rz) were presented by a bold star on top of a bracket.

Using the relative motion protocol, a significant effect was found for the factor implant type (p < 0.001), but not for digitizing resolution (p = 0.200). A follow up analysis was nonetheless performed, because a strong trend towards an interaction effect was observed (p = 0.067), revealed a significant difference within the factor digitizing resolution for HIP 1 (p < 0.001, Figure 6).

Due to the similar trends for migration measured between the zero relative prosthesis-bone motion (Figure 5) and relative prosthesis-bone motion (Figure 6), the values of the descriptive statistics reported below and in the tables (Table 1) will refer to the relative prosthesis-bone motion data only.

**Table 1:** Migration measured using MBRSA and the both RE models ATOS II and ATOS I respectively using the relative prothessis bone motion protocol (N = 10)

	**Model**		**In-plane**		**Out-of-plane**			**In-plane**
			**x [mm]**	**y [mm]**	**z [mm]**	**Rx [deg]**	**Ry [deg]**	**Rz [deg]**
**TIBIA**	ATOS II	Bias	.037	-.005	.048	.028	.007	.029
	ATOS I		.037	-.005	.024	.032	-.052	.035
	ATOS II	SD	.022	.019	.032	.047	.123	.028
	ATOS I		.023	.019	.030	.044	.187	.025
**FEMUR**	ATOS II	Bias	.032	-.001	-.042	-.055	-.060	.020
	ATOS I		.030	-.004	-.018	-.055	-.046	.011
	ATOS II	SD	.028	.012	.037	.033	.029	.036
	ATOS I		.028	.013	.027	.026	.028	.019
**HIP 1**	ATOS II	Bias	.012	-.017	-.025	-.030	-.449	.026
	ATOS I		.013	-.015	.015	-.035	.078	.029
	ATOS II	SD	.026	.023	.036	.038	.354	.015
	ATOS I		.025	.018	.058	.046	.298	.016
**HIP 2**	ATOS II	Bias	.021	-.007	-.017	-.079	-.138	.029
	ATOS I		.024	-.003	-.048	-.041	-.057	.034
	ATOS II	SD	.036	.018	.073	.110	.204	.051
	ATOS I		.047	.014	.181	.099	.330	.058

ATOS II data results out of a previois study using the same migration measurement protocol and measurement set-up for comparison [38]

Bias for translational and rotational motion for all the prosthesis components investigated ranged from -0.042 to 0.048 mm and -0.449 to 0.029 deg with the high resolution models (ATOS II) [38], and from -0.048 to 0.037 mm and -0.057 to 0.078 deg for the lower resolution RE models (ATOS I) respectively (Table 1). The worst rotational bias of −0.449 deg was observed with the high resolution models (ATOS II) [38], for the hip prosthesis with the largest length to width aspect ratio (HIP 1, Table 1 and Figure 6). Interestingly, the results indicate a *reduced* maximum bias for HIP 1 observed for rotational motion using RE models of *lower* digitizing quality (Table 1).

The SD as a measurement of data variability were observed in all cases for in-plane and for out-of-plane motion with a maximum SD of ± 0.073 mm and ± 0.354 deg observed when using the high resolution models (ATOS II) [38], and with a maximum SD ± 0.181 mm and ± 0.330 deg observed for the lower resolution RE models (ATOS I) respectively.

## Discussion

The dependence of the accuracy of MBRSA on RE model quality was investigated in an experimental phantom-model. For the measurement of in-plane implant motion, we observed no statistical loss of accuracy or precision when using lower resolution RE models. For out-of-plane motion, in particular for the total hip arthroplasty components, a dependence on RE model quality was observed. We thus reject the hypothesis that spatial resolution of the RE models does not affect the accuracy of the model-based RSA method. The effect of RE model resolution was different for the knee components investigated and for the hip components. Thus, maximum confidence-intervals for translation are marginally worse, and for rotation somewhat better for the knee components when using the lower resolution RE models (Table 1). Conversely, maximum confidence-intervals for translation are clearly worse for translation, and are marginally better for rotations using the lower resolution RE models. Nonetheless, the ranges of confidence intervals for translation observed were well within what has been reported in the literature (Table 2). Translational motion is of most interest to us, because it has been correlated to later aseptic loosening [14,15]. The largest data variability – bounded by 25^th^ and 75^th^ percentile in the box-plots (Figures 5D and 6D) and represented by SD (Table 1) – was in general observed for the total hip arthroplasty components for rotational motion about the out-of plane superior-inferior axis. Closer inspection of the data indicates that for the hip components, the RE models *of lower* digitizing quality generally lead to a wider data variability for out-of-plane motion: translational motion in the anterior-posterior direction (z, Figure 6E), as well as rotational motion about the superior-inferior axis (Ry, Figures 5D and 6D). The accuracy of the model-based method was in general, as expected, lower for out-of-plane than for in-plane motions. Thus, for both femoral stem total hip arthroplasty components (Hip 1, Hip 2), accuracy of MBRSA is lower for rotations about the superior-inferior axis, which represents out-of-plane motion along the long axis of the shaft of the prosthesis component (Figures 5D and 6D).

**Table 2:** Survey of RSA accuracy reported in the literature

**Author**	**Source**	**Ranges**	**Statistic**
*Selvik 1989*	prosthesis marker	0.02 to 0.45 mm	Max. error
		−0.02 to −0.19 deg	
*Karrholm 1989*	prosthesis marker	0.01 to 0.25 mm	Standard deviation
		0.03 to 0.6 deg	
*Valstar 2001*	surface model (computer aided design)	0.22 mm	Standard deviation
		0.52 deg	
*Valstar et al. 2002*	prosthesis marker	0.05 to 0.5 mm	95% confidence-interval
		0.15 to 1.15 deg	
*Kaptein et al. 2003*	surface model (RE)	0.14 mm	Max. standard deviation
		0.1 deg	

Remark: The indexed literature is a selection of the published literature.

Total hip arthroplasty components may require high(er) resolution RE models. The lower accuracy and precision of measurements of the total hip arthroplasty components for rotations about the superior-inferior direction is presumably the result of the nature of the model-based method [8,38]. Rotations about the axis of symmetry of axially-symmetric objects do not change the contour of the projected image to as large a degree as motion about a non-symmetric axes, and can thus not be detected as accurately using pose-estimation methods. We have however not systematically investigated the hypothesis that larger length-to-width aspect ratios alone are responsible for this difference, to do so would require a systematic study on simple representative geometries with differing aspect-rations. In the author’s opinion, prostheses components with significantly different geometries than those investigated herein should thus first be characterized in order to verify the suitability of the models to be used. The accuracy of measuring migration using the MBRSA method can to date only be determined my means of experimental phantom-model investigations. While similar results would be expected from other prosthesis components of similar geometry, materials and manufacturing tolerances, this must however still be verified. Besides the loss of accuracy previously observed for the femoral stem total hip arthroplasty components [8,38], the results of this current study further show higher variability of motion data when RE models of lesser quality are applied – in particular for rotational and translational migration in the out-of-plane directions.

## Conclusion

In summary, the results of the current study suggest that the MBRSA method delivers sufficient accuracy such that it could lead to wider application of RSA for the investigation of clinical implant fixation. In applications where more accuracy is required and in particular for implants of similar geometry as the hip-stems investigated, the quality of the RE model could become more meaningful. The MBRSA method is a promising approach, which enables the *in vivo* assessment of migration without the necessity of placing prosthesis markers. It furthermore allows migration measurement of prosthesis for which marker-based RSA could to date not be applied due to marker attachment and occlusion issues resulting from the typical geometry of such components. Nonetheless, further studies will be necessary to investigate the applicability of MBRSA to specific prosthesis components and designs before the method is used to investigate such prostheses in a clinical setting.

## Abbreviations

RSA: Roentgen Stereophotogrammetric Analysis; MBRSA: Model-based Roentgen Stereophotogrammetric Analysis; CAD: Computer aided design; RE: Reverse engineering; IIPM: Iterative inverse perspective matching; SD: Standard deviation.

## Competing interests

The authors declare that they have no competing interests.

## Authors’ contributions

The following authors have designed the study (FS, CH), performed the experiments, gathered and analyzed the data (FS, JE), written the initial draft (FS, CH, JE, BK, HW), and ensured the accuracy of the data and analysis (FS, CH, JE, BK, HW). All authors read and approved the final manuscript.

## Authors’ information

J. Emmerich, B.L. Kaptein, H. Windhagen and C. Hurschler are co-authors.
